# Social Media and Mobile Apps for Health Promotion in Australian Indigenous Populations: Scoping Review

**DOI:** 10.2196/jmir.3614

**Published:** 2014-12-10

**Authors:** Carl Brusse, Karen Gardner, Daniel McAullay, Michelle Dowden

**Affiliations:** ^1^Australian Primary Health Care Research InstituteResearch School of Population HealthAustralian National UniversityCanberraAustralia; ^2^Independant consultantDarwinAustralia

**Keywords:** health promotion, indigenous health, eHealth

## Abstract

**Background:**

Health promotion organizations are increasingly embracing social media technologies to engage end users in a more interactive way and to widely disseminate their messages with the aim of improving health outcomes. However, such technologies are still in their early stages of development and, thus, evidence of their efficacy is limited.

**Objective:**

The study aimed to provide a current overview of the evidence surrounding consumer-use social media and mobile software apps for health promotion interventions, with a particular focus on the Australian context and on health promotion targeted toward an Indigenous audience. Specifically, our research questions were: (1) What is the peer-reviewed evidence of benefit for social media and mobile technologies used in health promotion, intervention, self-management, and health service delivery, with regard to smoking cessation, sexual health, and otitis media? and (2) What social media and mobile software have been used in Indigenous-focused health promotion interventions in Australia with respect to smoking cessation, sexual health, or otitis media, and what is the evidence of their effectiveness and benefit?

**Methods:**

We conducted a scoping study of peer-reviewed evidence for the effectiveness of social media and mobile technologies in health promotion (globally) with respect to smoking cessation, sexual health, and otitis media. A scoping review was also conducted for Australian uses of social media to reach Indigenous Australians and mobile apps produced by Australian health bodies, again with respect to these three areas.

**Results:**

The review identified 17 intervention studies and seven systematic reviews that met inclusion criteria, which showed limited evidence of benefit from these interventions. We also found five Australian projects with significant social media health components targeting the Indigenous Australian population for health promotion purposes, and four mobile software apps that met inclusion criteria. No evidence of benefit was found for these projects.

**Conclusions:**

Although social media technologies have the unique capacity to reach Indigenous Australians as well as other underserved populations because of their wide and instant disseminability, evidence of their capacity to do so is limited. Current interventions are neither evidence-based nor widely adopted. Health promotion organizations need to gain a more thorough understanding of their technologies, who engages with them, why they engage with them, and how, in order to be able to create successful social media projects.

## Introduction

### Health Promotion, Health Outcomes, and Technology

One key strategy used in health promotion interventions is to engage a target audience among the general public, with the goal of improving health outcomes by influencing the behavior of that audience. The intended health concerns and behavioral goals will vary from project to project and the target audience might be a narrow demographic or broader and more general, but the basic nature of this particular aspect of health promotion is the same: a non-commercial marketing exercise in the name of a greater social good. This subset of health promotion can therefore be seen as a species of “social marketing”.

Understood as social marketing, health promotion interventions can be, should be, and indeed are being taken beyond traditional public service messages and familiar advertising campaigns [1-3]. Marketing of all forms has become increasingly sophisticated in the online era, with new media modalities and analytics enabling more targeted approaches to both commercial and social marketing. Among the numerous health promotion projects and engagement strategies being explored, new media technologies such as social media and mobile phone/tablet apps have attracted a great deal of interest from public health organizations and researchers [3,4].

The potential for using new media to achieve improved health outcomes is broader than social marketing alone. The interactive nature and the personal intimacy of these new technologies for an “always online” health consumer allows for their use in self-management and even in the delivery of some health services. However, in part because these techniques are in their infancy (and for other reasons that shall be discussed), early evidence of effectiveness and benefit is lacking.

The research reported here is intended to provide a current overview of the evidence surrounding *consumer-use* social media and mobile software (apps) for health promotion interventions, with a particular focus on the Australian context and on health promotion targeted toward an Indigenous audience. This intent and focus directed three component scoping surveys: (1) a survey of the uses of social media to reach Indigenous Australians for health promotion, (2) a survey of Australian health promotion/social marketing projects that make use of social media and mobile apps, and (3) a scoping study of peer-reviewed evidence for the effectiveness of social media and mobile technologies in health promotion, globally.

### The Internet and Social Media

It has been 10 years since the average time that young Americans spent online exceeded the time they spent watching TV; a statistic that the general population in developed countries has gradually been catching up with. The time spent on online social media has ballooned since the introduction of dedicated social media platforms in 2004-2007 and now amounts to at least 20% of all time spent online, with that proportion again much higher in the youth demographic. Publically available Web traffic measurements now rank the leading social media domain, Facebook.com, as either the first or second most popular domain in Australia, marginally ahead of or behind Google.com domains, depending on the methodology used. Indeed, some industry watchers have even speculated that the rapidly increasing proportion of online content being created, shared, and consumed within social media “walled gardens” may be altering the fundamental character of the Web, its openness, and how it is used.

For the moment though, and for the purposes of social marketing, including health promotion, the upshot of these changes is simple: social media is increasingly where the eyeballs are (not to mention fingertips). If there is a message to present or content to deliver, then establishing a social media presence represents both an opportunity and increasingly a requirement.

Social media also holds attractions for the social marketer beyond mere reach. For example, minimal resources are required to create a basic online presence in social media platforms such as Facebook: a Facebook page-making platform can generate a ready receptacle for publishing or publicizing updatable content, from simple comments and posts to images, video, and interactive media. The most salient and alluring promises of social media however (for health promotion and other social marketing) come with the social aspects of social media.

The “social” in social media refers to method and modality, rather than to goals or objectives as in “social marketing”. The activities that constitute social media—users forming social connections and carrying out social interactions via the medium of social media platforms—can be seen as the extension of natural human social networking into online spaces. Social media users add or re-transmit pictorial, textual, or multimedia content that they find interesting, sharing it with peers, circles of contacts, or the Web at large. Social media therefore combines the reach of mass media, but is integrated into and driven by the small-scale social networking of everyday people. The Holy Grail for any social marketer is for their message to be picked up and transmitted widely via online social networks (to “go viral”) and thereby radically magnify its reach and effect.

But social media is complex. First, because it is driven by user interest, it is up to the users to decide whether any particular content is taken up and re-transmitted (and with what framing, eg, serious or sarcastic). Second, the modalities of social media are themselves complex. Some social media is carried out via secondary features on specialized content-focused platforms (eg, YouTube.com), some within rich and freestanding feature-packages (such as with Facebook.com), and some via minimalist messaging and micro-blogging services (eg, Twitter.com). And the social media “workflow” of users can involve the mixing and matching of social media functions, online content, and other messaging and publishing tools (such as instant messaging/chat, Web forums, blogs, webpage comments, and even email) within a sophisticated ecosystem of online social behavior. Broad characterizations and analyses of social media usage must therefore be treated with caution, because social media is defined by use and function rather than by form or design.

For the health promotion practitioner, social media is therefore a complex beast with great potential for both success and failure—a way of starting and carrying on an actual conversation with the target audience (as long as their attention is acquired and retained), which has the potential to take on a life of its own (for better or for worse) or simply be ignored.

### Mobile Technology

A second major trend in recent online behavior is that more and more “eyeball time” takes place on mobile devices outside the Web browser. In 2011 (only 4 years after the introduction of the first iPhone), sales of mobile phones overtook those of traditional desktop and laptop computers and the expectation is that tablet devices will do the same within 2 years [5]. The rapid uptake of these mobile devices has driven a whole new class of lightweight software—mobile apps—to suit compact, touch-screen interfaces and to integrate with telephony, GPS, and sensor features included in the always-on, battery-dependent mobile hardware.

Well-written mobile apps are, in effect, pre-packaged caches of interactive content, installed on devices that users carry about with them. Whereas traditional websites for example require a constant, reliable Internet connection to be accessed, users have mobile apps on their phone at all times, including when Internet access is spotty or when they’ve run out of data.

Mobile apps are therefore a tantalizing target for health promotion purposes—and for similar reasons to social media. The uptake of social media and mobile apps is user-driven: meaning they are interventions that can potentially be disseminated much more widely than a traditional intervention where subjects are individually recruited. Furthermore, the “buy-in” effect is potentially very significant for apps that successfully make it onto mobile devices and actually get used. As with social media projects then, the trick would be to design apps that are attractive and accessible to the target population, as well as constituting effective interventions.

### Indigenous Use of Social Media and Mobile Technology in Australia

For the purposes of our review, the target population of interest is Indigenous Australians: a traditionally underserved population with a high prevalence of public health issues, with particular areas of concern such as smoking cessation, sexual health, and otitis media commonly identified by health care workers.

International studies have suggested that mobile devices can help traditionally underserved populations by leapfrogging economic and infrastructural bottlenecks—bringing connectivity to individuals in remote or underserved communities without having to wait for access to more traditional (and expensive) residential Internet or computer use [3]. But Indigenous cultures in Australia also deeply embed practices of social relatedness, kinship, and identity with specific priorities for communication and social interconnection despite the remoteness of some of these communities. As might be expected then, there is a high penetration of mobile phone usage among Indigenous Australians including those in rural and remote communities. Mobile phones are by far the most used technology among adolescents [6], surpassing TV, video games, and other forms of Internet access. In the past 5 years, affordable mobile phones with camera and messaging functions have spawned a “mobile phone culture” in some remote areas, where messages, pictures, and video clips flow freely among and between communities, often in culturally unique and creative ways [7].

The questions for health promotion in this area therefore are: what is being done, what can be done, and what should be done to leverage this rapid uptake of mobile and social media technologies among Indigenous Australians for health promotion purposes? Can mobile or social media technologies be effective for health promotion in such communities and, if so, then how? Questions such as these shaped the focus of this initial scoping research.

## Methods

### Overview

In consultation with a reference group drawn from the Australian Primary Health Care Research Institute (APHCRI) and the Aboriginal Health Council of Western Australia (AHCWA), the research reported in this study aimed to answer the following two research questions: (1) Literature: What is the peer-reviewed evidence of benefit for social media and mobile technologies used in health promotion, intervention, self-management, and health service delivery, with regard to smoking cessation, sexual health, and otitis media? and (2) Projects: What social media and mobile software have been used in Indigenous-focused health promotion interventions in Australia with respect to smoking cessation, sexual health, or otitis media, and what is the evidence of their effectiveness and benefit?

### Peer-Reviewed Literature

The scoping study contained two review components. The first was a review of recent publications in peer-reviewed literature, following the framework outlined in Arksey (2003), with the intent to summarize and disseminate research findings and identify gaps in the existing literature. We included both original studies and systematic reviews, looking for evidence of benefit associated with relevant eHealth interventions. The literature search was conducted in November 2013 using Ovid/MEDLINE, searching for publications since 2011 (inclusive) using the following search strategy:

1. exp Health Promotion/ or Health Communication/ or Health Education/ or Social Marketing/ or Health Literacy/ or Preventive Medicine/ or Preventive Health Services/ or Primary Prevention/ or Delivery of Health Care/ or Health Services Accessibility/ or Sexual Behavior/ or Smoking Cessation/ or Smoking/ or Otitis media/
2. exp Social Media/ or Cellular Phone/ or Text Messaging/ or computers, handheld/
3. (facebook or social media or social networking or SMS or text messag$ or smart phone or smartphone or iPhone or iPad or new media or “Web 2.0”).af.
4. (facebook or social media or social networking or SMS or text messag$ or smart phone or smartphone or iPhone or iPad or new media or “Web 2.0”).m_titl.
5. 2 or 3 or 4
6. 1 and 5

Both primary studies and systematic reviews were collected and assessed by title and abstract for further review.

To be included, studies or reviews had to report on the impact of a health promotion intervention on health or behavioral outcomes, for example: smoking status, sexual health behavior, or improvements in relevant knowledge, attitudes, or intent. Qualitative and quantitative studies were included. All target populations (Indigenous or otherwise) were included.

Included studies used social media (on any platform) or mobile phone software or features (excluding voice features). Automated SMS (short message service) text messaging-only interventions were included on the basis that the basic character of such interventions could reasonably be reproduced using mobile software; other mobile, non-smartphone interventions were included on a similar basis of broad similarity.

Studies of direct communication methods between health services and patients or technological extensions of normal health service activities (eg, reminders or communication of test results) were excluded. Website-delivered interventions (with no social media component) were excluded. Speculative articles and publications not reporting empirical results were excluded. Study protocols and feasibility studies reporting only on acceptability of interventions were also excluded.

Systematic reviews were examined for scope of search, summative evidence reported, and conclusions regarding evidence according to the criteria of this scoping study (outlined above). Cited articles in systematic reviews were assessed for secondary inclusion in the same manner as primary studies.

Data were extracted into a template that recorded the study focus (technological modality and disease focus), method and study population, and evidence of impact or outcome. Multiple publications from the same study were grouped together. Evidence of impact or outcome was broken down according to four broad domains of improvement: knowledge or awareness of relevant health information, health-related attitudes (eg, self-reported behavioral intent or measures of self-efficacy), health-related behaviors, and health outcomes.

### Australian Projects

The second component of the scoping exercise looked for (1) publically available mobile apps projects created or promoted by Australian health bodies (including government departments and agencies, Indigenous health organizations, and health promotion agencies) and targeted at Indigenous populations, and (2) social media projects and initiatives created or promoted by Australian health bodies (including government departments and agencies, Indigenous health organizations, and health promotion agencies) and targeted at Indigenous populations.

The primary method used was domain-restricted searches of the websites of these organizations, using the Google search engine with the search terms: “iphone”, “android”, “social media”, and “Facebook”. The term “site:[domain]” was included to search only indexed pages within the domains in question. Descriptions of Indigenous public health projects maintained at the Centre for Excellence in Indigenous Tobacco Control [8] and the Australian Indigenous Health Infonet [9] were manually searched for mentions of social media and mobile software app components. Android and iOS app stores were searched using the search terms “Indigenous”, “Aboriginal”, “Smoking”, and “Health”.

Apps designed to be used by clinicians or other health professionals (eg, medical reference guides or clinical advice support apps) were excluded from the study. Inclusion criteria for both mobile software and social media apps were broad. Any app that appeared to be aimed at an Indigenous audience or which reported intentions in that regard was included. Any use of social media or mobile software apps within larger health promotion projects was included.

In all included cases, we also looked for publically available evaluations of these projects and evidence of benefit, as well as rudimentary measures of reach and uptake including number of “likes” or downloads on the relevant social media platforms and app stores. Process evaluations were not included.

## Results

### Peer-Reviewed Literature

Our search returned 560 results, which were screened by title and abstract, of which 28 were selected for further examination. Of these, 10 primary intervention studies met our inclusion criteria: six for smoking cessation, four for sexual health, and none for otitis media. Also included were seven systematic reviews: four of a general disease focus, which contained evidence relevant to our inclusion criteria, two specific to smoking cessation, and one specific to sexual health. From these review publications, we secondarily included a further eight publications specific to smoking (drawn from six discrete studies/data sets) and two specific to sexual health.

Two of our nine primary inclusions were also present in review publications; there was one secondary inclusion published during the interval for primary inclusion but not found by our search strategy.

Systematic reviews of a general focus are summarized in Table 1, with all smoking-specific studies in Table 2, and all sexual health-specific studies in Table 3. We found no studies focusing on otitis media that met the inclusion criteria.

One smoking cessation study and two sexual health intervention studies demonstrated some evidence of benefit with both knowledge of relevant health information and health-related attitudes, however none of these three demonstrated improvement in health-related behaviors. Statistically significant improvement in health-related behaviors was reported in four smoking cessation studies. Evidence for behavioral benefit after sexual health interventions was small or tentative and no studies attempted to measure benefits for direct health outcomes.

### Australian Projects

We found five projects with significant social media health components targeted at the Indigenous Australian population for health promotion purposes. Of these, four primarily targeted smoking cessation and two targeted sexual health and/or behavior. We found no publically available evaluations of these projects that included evidence beyond that for process evaluation. Available metrics of reach and impact included Facebook page “likes” ranging between 383 and 2694 users, and 17,143 views in the case of one YouTube video.

We also found 29 examples of mobile phone apps produced, funded, or promoted by Australian health promotion bodies, four of which were identifiably for health promotion purposes targeting the Indigenous population, including three concerned with smoking cessation and behavior and one for otitis media awareness. We found no publically available evaluations of any of these apps. Available metrics of reach and impact from app stores suggested less than 100 installations for the least installed app and more than 5000 for the most popular, which was rated at 4.1/5 and 4.5/5 on the two most popular app stores. There were too few reviews to draw statistical conclusions in the case of the three apps most closely targeted at an Indigenous audience. These results are detailed in Table 4.

**Table 1:** Summary of general relevance reviews.

Publication	Study summary	Findings/outcomes
Yeager, V & Menachemi, N, 2011. Text messaging in health care: a systematic review of impact studies. [10]	Systematic review of text messaging use in health care; publications prior to 2010.	N=127 articles identified for consideration, n=61 reviewed with n=24 public health related. Publications began in 2003. 7 secondary inclusions: [11-17]
Vodopivec-Jamsek, V et al, 2012. Mobile phone messaging for preventive health care. Cochrane Database of Systematic Reviews. [18]	Systematic review of SMS^a^/mobile messaging – all primary prevention uses, effect on health status and health behavior outcomes. Carried out June 2009.	N=3937 citations, n=4 included interventions on health behaviors (out of 31 studies reviewed). “Very limited evidence”, high quality evidence for smoking cessation intervention only. 1 secondary inclusion: [12]
Chou, WS et al, 2013. Web 2.0 for Health Promotion: Reviewing the Current Evidence. [3]	Systematic review of social media use in health settings. Reviews and commentaries, descriptive studies, intervention studies.	N=1258 citations, n=34 included intervention studies. Only 10 small and/or pilot studies found where social media was used in interventions. 1 secondary inclusion: [19]
Free, C et al, 2013. The Effectiveness of Mobile-Health Technology-Based Health Behaviour Change or Disease Management Interventions for Health Care Consumers: A Systematic Review. [4]	Systematic review of literature 1990 – September 2010. All uses of mobile Meta-analysis including assessment of bias risk.	N=36,314 citations, n=26 included interventions on health behaviors (n=75 total inclusions). SMS-smoking cessation trials (only) are shown to be effective in the meta-analysis. 5 secondary inclusions: [12,13,20-22]

^a^SMS: short message service

**Table 2:** Summary of smoking cessation reviews.

Publication		Study summary	Findings/outcomes (K=knowledge, A=attitude, B=behavior, H=health)
**Primary studies**			
	Free, C et al, 2011. Smoking cessation support delivered via mobile phone text messaging (txt2stop): a single-blind, randomised trial. [21]	RCT^a^of text messaging program for smoking cessation. N=5800 smokers wishing to quit, UK, 2007-2009. Abstinence biochemically verified.	B - Positive Significant increase in verified abstinence at 6 months, 10.7% intervention vs 4.9% control. Also included via: [4,23]
	Reitzel, L et al, 2011. The efficacy of computer-delivered treatment for smoking cessation [24], also: Wetter et al. (2011) A randomized clinical trial of a palmtop computer-delivered treatment for smoking relapse prevention among women. [25]	RCT of palmtop-delivered intervention. USA, 1999-2003. N=303 adult smokers, randomized into palmtop intervention and regular intervention.	B - Negative No significant differences in outcomes between intervention groups.
	Whittaker, R et al, 2011. A theory-based video messaging mobile phone intervention for smoking cessation: randomized controlled trial. [26]	RCT of SMS^b^and mobile video content (complex) for smoking cessation. New Zealand, 2007 & 2009. N=226 adults recruited via advertising.	B - Negative Not able to demonstrate statistically significant effect for complex/tailored message and video regime over control. Failed to reach sample size. Also included via: [23]
	Naughton, F et al, 2012. Randomized controlled trial evaluation of a tailored leaflet and SMS text message self-help intervention for pregnant smokers (MiQuit). [27]	RCT of text messaging as part of support package for smoking cessation. UK, 2008-2009. N=207 pregnant smokers. Intervention of tailored literature and intervention SMS vs standard self-help literature and evaluation SMS.	K - Positive, A - Positive, B - Negative Positive effects on “key intervention targets: self-efficacy, harm beliefs, and determination to quit”, and increased likelihood of setting quit date. Self-reported and biochemically verified abstinence rate difference not statistically significant. Study was “not powered to detect a group difference on smoking outcomes”.
	Ybarra, M et al, 2012. A text messaging-based smoking cessation program for adult smokers: randomized controlled trial. [28]	RCT of text messaging for smoking cessation. Turkey. N=151 adult smokers wanting to quit. Randomized into text messaging and brochure groups, 3-month follow-up.	B - Mostly negative Overall biochemically verified 3-month cessation trends not significantly higher in intervention group, except for women where 5 female participants quit (15%), significantly more than in control (0).
**Systematic reviews**			
	Chen, Y et al, 2012. Effectiveness and cost-effectiveness of computer and other electronic aids for smoking cessation: a systematic review and network meta-analysis. [29]	Broad systematic review and network meta-analysis of all “computer and electronic aids” for smoking cessation. Inclusions up to 2009.	B - Positive/tentative N=3969 citations, 77 papers from 60 RCTs included. Effectiveness review: studied aids increase likelihood of cessation “but the effect is small”. Likely to be cost effective for smokers seeking to quit. Not able to determine what form of electronic aids are more effective. 5 secondary inclusions: [12-15,20]
	Whittaker, R et al, 2012. Mobile phone-based interventions for smoking cessation. [23]	Narrow Cochrane review of long term (6 month+) studies of text messaging interventions for smoking cessation, carried out May 2012.	B - Positive/mixed 5 studies included out of 17 reviewed. Three used the same “text2quit” SMS intervention adapted for different populations and contexts (Rogers 2005; Free 2009; Free 2011). 4 secondary inclusions: [12,20,21,26]
**Secondary inclusions from systematic reviews**			
	Obermayer, JL et al, 2004. College Smoking-Cessation Using Cell Phone Text Messaging. [16]	Cohort study, N=46 young adult smokers, Web and text messaging “quit program” intervention for cessation.	B: Tentative Small, uncontrolled pilot study with modest outcome measures. 43% participants attempted to quit for at least 24h and 17% had been abstinent for 1 week after 6 weeks. Included via [10].
	Rodgers et al (2005) Do u smoke after txt? Results of a randomised trial of smoking cessation using mobile phone text messaging [12], and: Bramley et al (2005) Smoking cessation using mobile phone text messaging is as effective in Maori as non-Maori. [11]	RCT, N=1705 adult New Zealanders, 26-week text messaging intervention for cessation.	B: Positive More participants had quit at 6 weeks in the intervention compared to control group: 239 (28%) vs 109 (13%). Intervention as effective in Maori as non-Maori. Long-term results compromised by incomplete follow-up and over-reporting of quit rate. Included via [4,10,18,23,29].
	Brendryen and Kraft (2008) Happy Ending: a randomized controlled trial of a digital multi-media smoking cessation intervention [14], and: Brendryen et al (2008) A Digital Smoking Cessation Program Delivered Through Internet and Cell Phone Without Nicotine Replacement (Happy Ending): Randomized Controlled Trial. [15]	RCT, Norway. N=396 smokers, 56 week automated text message, email, and Web-based smoking cessation intervention.	B: Positive Statistically significantly higher repeated point abstinence rates (at 1, 3, 6 and 12 months) than control participants (22.3% vs 13.1%). Included via [10,29].
	Whittaker, R et al, 2008. A Multimedia Mobile Phone-Based Youth Smoking Cessation Intervention: Findings From Content Development and Piloting Studies. [17]	Feasibility study and uncontrolled pilot study. New Zealand. N=15 young smokers, 4-week video messaging program for smoking cessation.	B - Tentative Mostly a feasibility study. Positive feedback from pilot group suggesting improved smoking abstinence (not verified). Included via [10].
	Haug, S et al., 2009. Continuous individual support of smoking cessation using text messaging: A pilot experimental study. [13]	RCT of text messaging for smoking cessation. Germany. N=174 adults, weekly text message question followed by 1 or 3 feedback text messages and helpline.	B - Negative No significant differences found in smoking variables around abstinence. Primarily a feasibility study. Included via [4,10,29].
	Free, C et al., 2009. Txt2stop: a pilot randomised controlled trial of mobile phone-based smoking cessation support. [20]	Pilot of RCT of text messaging program for smoking cessation [21]. UK. N=200 smokers wishing to quit. Abstinence biochemically verified.	B - Positive Abstinence rates at 4 weeks significantly better for intervention group: 26% vs 12%. Included via [4,23,29].

^a^RCT: randomized controlled trial

^b^SMS: short message service

**Table 3:** Summary of sexual health reviews.

Publication		Study Summary	Findings/outcomes (K=knowledge, A=attitude, B=behavior, H=health)
**Primary inclusions**			
	Juzang, I et al, 2011. A pilot programme using mobile phones for HIV prevention. [30]	Pilot/feasibility trial of text messaging for sexual health knowledge and behavior. USA. N=60 young black men. 12 week trial, intervention received sex-ed messages, control received nutrition messages.	K - Positive, A - Positive, B - Negative Participants showed trends in increased monogamy at follow-up compared to controls. Awareness of sexual health significantly higher in the intervention group. Condom norms significantly higher for control. No differences in proportion of protected sex acts.
	Gold, J et al, 2011. A randomised controlled trial using mobile advertising to promote safer sex and sun safety to young people. [31]	RCT^a^of SMS^b^advertising for sexual health behavior. Victoria, Australia, 2009. N=7606 16-29 y/o mobile advertising subscribers randomized into “sex” or “sun” groups. Baseline and follow-up mobile phone questionnaire.	B - Mixed Significant OR (self-reported) for less sexually risky behavior (eg fewer sexual partners, always using condoms). Increased STI testing not found. Only 10% of participants completed any surveys, only 151 completed both baseline and follow-up surveys. Authors cite “challenges experienced during intervention implementation”.
	Bull, S et al, 2012. Social media-delivered sexual health intervention: a cluster randomized controlled trial. [32]	Cluster RCT trial of Facebook messages for condom use. USA, October 2010-May 2011. N=1578 self/peer-recruited enrollees to a Facebook page with sexual health messages. Condom use surveyed at 2 and 6 months.	A - Negative, B - Positive/Tentative Small differences in condom use seen at 2 months (eg, 63% vs 57%, *P*=.03), no effects seen at 6 months. No effect on self-efficacy or norms.
	Reback, C et al, 2012. Text messaging reduces HIV risk behaviors among methamphetamine-using men who have sex with men. [33]	Non-controlled study of text messages targeting risky sexual behavior and drug use. USA October 2008-May 2009. N=52 methamphetamine-using men who have sex with men. Text messages over 2 weeks.	B - Tentative Self-report behavior significantly improved at 2 months. Biochemical verification of self-reported outcomes not statistically significant. Not controlled, baseline follow up only.
**Systematic reviews**			
	Guse, K et al, 2012. Interventions using new digital media to improve adolescent sexual health: a systematic review. [34]	Broad review of interactive digital media (new media) for adolescent sexual health.	B - Positive/mixed N=942 citations, 10 included. Mostly Web-delivered interactive interventions, three showing significant impact in dangerous sexual behaviors. 1 secondary inclusion: [19]
**Secondary inclusions**			
	Moreno, MA et al, 2009. Reducing at-risk adolescents’ display of risk behavior on a social networking Web site: a randomized controlled pilot intervention trial. [19]	RCT of intervention to alter online displays of risky behavior. N=190 18-20 year olds with MySpace profiles. Intervention group sent single message from clinician.	B - Tentative/negative Online displays of risky sexual behavior slightly reduced 3 months after intervention, no significant change in other measures. Only online behaviors studied. Included via: [3,34]
	Lim, MSC et al, 2012. Impact of text and email messaging on the sexual health of young people: a randomised controlled trial. [22]	RCT on 12-month program of email and text messages to young adults. S&P: N=994 recruited at music festival. Self-reported behavior and knowledge via survey at recruitment, 3, 6, and 12 months.	K - Positive, A - Positive, B - Negative At 12 months, STI knowledge was higher in the intervention. Women (but not men) in the intervention group were more likely to have had an STI test or discuss sexual health with a clinician. No significant impact on condom use. Included via: [4]

^a^RCT: randomized controlled trial

^b^SMS: short message service

**Table 4:** Australian apps and social media programs with Indigenous focus.

Name of app or campaign	Organization	Description	Evaluation or evidence of reach / impact
Stickin’ it up the smokes	Aboriginal Health Council of South Australia	Social Media – Smoking. Social marketing campaign with prominent Facebook page, targeting smoking cessation/abstinence for young Aboriginal women.	Reach/impact evaluation not available at time of study. Facebook page has 1274 likes, 0-19 likes per post.
It’s your choice! Have a voice! rights, respect, responsibility 2013	Aboriginal Health and Medical Research Council	Social Media – Sexual Health. Publicity campaign partnered with Indigenous Hip Hop Projects (IHHP), “bringing a state-wide arts based campaign to empower and educate Aboriginal adolescents to make informed choices about sexual and reproductive health and understand the negative impacts of alcohol and other drugs”. Facebook page included as part of broader campaign.	No publically available evaluation found. Facebook page has 2694 likes, 0-54 likes per post.
Kasa por yarn	Funded by Queensland Health.	Social Media – Sexual Health. Audio/video drama with YouTube and Facebook presence, funded by Queensland Health. Two series on Torres Strait Radio 4MW in 2011 and 2012. Storylines and characters were locally developed on Thursday Island.	No publically available evaluation found. Facebook page active 2011-2012, currently has 2639 likes. YouTube channel’s 42 videos have between 88 and 1936 views.
Rewrite your story	Nunkuwarrin Yunti of South Australia Inc.	Social Media – Smoking. Web–based campaign focused on social media, launched January 2013. Includes sophisticated website with interactive “pledge” feature, Facebook page, and YouTube channel hosting personal “stories” about smoking and smoking cessation.	No publically available evaluation found. Main website includes 402 “pledges and stories”. Facebook page active 2011-present, currently has 443 likes. YouTube channel’s 20 videos have between 8 and 1793 views.
NoSmokes.com.au	Menzies School of Health Research	Social Media/Mobile Software – Smoking. Suite of online projects /experiments designed for use by Aboriginal and Torres Strait Islander people, including mobile software, videos, and online games. Hosted from dedicated website, Facebook page, and YouTube channel.	Only focus-group/process evaluation currently available. Facebook page active 2010 to present has 383 likes, YouTube channel’s 33 videos have between 9 and 17,143 views.
Hip Hop Dance-Off	Menzies School of Health Research	Mobile Software – Smoking. Part of “No Smokes” suite of eHealth projects.	10 ratings on iTunes store, 1000-5000 installs on Google Play store.
No Smokes/So you think you can Quit?	Menzies School of Health Research	Mobile Software – Smoking. Part of “No Smokes” suite of eHealth projects. App available for iPhone, iPad, and Android.	9 ratings on iTunes store, 10-50 installs on Google Play store.
Quit for you, quit for two	Commonwealth Department of Health	Mobile Software - Smoking. Mobile app, part of government advertising campaign intended to encourage mothers from a “diverse background” to quit smoking. Includes tracker/educational component for baby progress and money saved, and an animated baby character will play games, assist with timing breathing, etc. Includes Quitline connection and other support options.	5000-10,000 installs on Google Play store with 21 ratings at 4.1/5, 6 ratings on iTunes store at 4.5/5.
Talking Book/Care for Kids Ears	Commonwealth Department of Health	Mobile Software – Otitis Media. Basic ear health information presented in style of an interactive children’s book, read in English or many Indigenous languages.	100-200 installs on Google Play store, insufficient ratings on iTunes store.

## Discussion

### Summary of Findings

With our survey of the relevant peer-reviewed literature, our main finding was that the evidence of benefit for social media and mobile apps in the specific areas of interest for Indigenous health in Australia is, for the most part, tentative and scattered. The most compelling body of evidence, relevant to this study, comes from a series of international studies into text messaging for smoking cessation. The main positive results here begin with a 2005 study in New Zealand [11,12], which was then adapted for further studies in the United Kingdom [20,21] and Turkey [28]. While these results are promising for messaging-based encouragement programs (and apps that might mimic such functionality via mobile software), this is largely an isolated body of research on messaging interventions specifically, and the transferability of such results from the context of communication to purely automated applications is not clear.

In fact, no peer-reviewed research was found at all for mobile software apps as such and only a single study was found where a social media intervention was linked to a significant (though small) change in behaviors that were *directly* health-relevant [32]. The vast majority of included studies that examined evidence of benefit studied the use of SMS text or similar messaging services to provide reminders, education, or preventive health messages. The key technologies of interest with the lowest barriers of entry and highest potential for dissemination—mobile app software and social media—do not yet appear to have a significant body of peer-reviewed evidence examining their effectiveness.

Our scoping study found four extant uses of social media in specific public health projects targeting an Indigenous audience, in most cases utilizing ready-made platforms such as Facebook and YouTube. The four mobile software apps that met the inclusion criteria included two produced by the Australian Commonwealth Government, and two produced as part of a broader research effort into eHealth for smoking cessation: the “No Smokes” project, which is also included in Table 4.

Among these projects we found a mix of strategies, including health promotion content produced with participation from members of the intended audience (eg, Kasa por yarn), and social media use as an addition to a large health campaign touring schools and communities (It’s your choice! Have a voice!). In contrast, the projects from “No Smokes” and “Rewrite your story” appeared to rely on a more top-down or Web-based approach to dissemination. The two Commonwealth-funded mobile software projects also differed in how narrowly they focused on the Indigenous population, with “Care for kids’ ears” explicitly aimed at Indigenous children and caregivers, but the more widely used “Quit for you, quit for two” app appealing to a much broader audience and being only loosely tied to the government’s target to reduce the Indigenous smoking rate.

### The Limited Scope of Evidence

Of the text messaging studies, a significant number have shown robust results for interventions to aid in smoking cessation for smokers trying to quit, either on their own or as part of a broader “quit smoking” program—a result confirmed in a recent systematic review and meta-analysis [4]. There is less evidence however for text messaging interventions for sexual health and other areas, though some studies here are also promising.

However, this imbalance of the evidence in favor of text messaging interventions is no doubt partly due to the relative maturity and ubiquity of this mobile technology (compared to mobile app software and social media), and may also speak to specific difficulties with conducting research on the newer technologies and/or publishing such research in peer-reviewed journals.

In any case, the lack of peer-reviewed or publically available evidence for interactive social media and mobile software projects is especially curious given the number of such projects being implemented in Australia alone.

We suggest that there are three distinct issues that may help explain this lack of evidence.

### Study Limitations and the Terminology Problem

One distinguishing feature of research in this area is that the language itself can be fragmented and ambiguous. For example, the term “eHealth” is one of the oldest and most well-established, but ambiguously refers to many different kinds of communications technology: technologies for connecting consumers and health services, for interconnecting health services, for training purposes, or simply as an extension of eCommerce (ie, as advertising/marketing/sales systems for commercial health services [35]).

This ambiguity within the research literature alone has itself been an object of study and been recognized in systematic reviews, as summarized in a 2005 review by Oh et al: “As with most neologisms, the precise meaning of eHealth varied with the context in which the term was used. Nevertheless, it has been fairly well understood, and is now widely used by many academic institutions, professional bodies, and funding organizations. We recognized the impossibility of finding an universally acceptable, universally applicable formal definition, yet felt that a clearer understanding of the term could be achieved by reviewing the range of proposed meanings” [36].

The result of this phenomenon is that multiple definitions and formalizations have been used by researchers to try to capture only partially overlapping concepts, with little or no systematic approach between those researchers and consumers, health services, and content creators. This has also seen new terms being coined both in the health context (eg, mHealth for mobile phone-based technology use) and in the broader community to describe new trends in the overall technology marketplace (eg, Web 2.0). So the terms researchers might seek to use and their referential extension over time can be extremely volatile. Furthermore, the rapid growth and aggressive use of branding in this area of commerce and technology has created what might be called the Hoover, Xerox, or iPod effect: where brand names and proprietary eponyms like “Facebook” dominate (and in some cases predate) the use of generic terms like “social media”. In many of the titles and abstracts considered in this study, the brand names of technologies used (eg, Facebook) were the only text-searchable identifier of relevance, suggesting that standardized nomenclature in research is struggling to catch up with its subject matter.

It is beyond the scope of this study to consider whether this terminologically heterogeneous research environment implies genuine conceptual fragmentation, however, at a practical level at least, it certainly means that search strategies must either focus narrowly or aim for being thorough at the expense of subsequent workload. There are no good MeSH terms that capture the area of interest presented in this review and no simple, stable language for keyword text searches. For example, one recent systematic review used a search strategy with keywords that took up 2 pages and returned over 26,000 electronic citations for review, out of which 75 were eventually deemed relevant [4]. In terms of workload alone, this represents a formidable challenge for researchers.

### The Measures Problem

A second issue is that the nature of the technology being used does not allow for clear implementation measures or clear outcome measures. For example, social media, websites, and app software are “use on demand” services in that they are engaged with by users to varying degrees and under a variety of conditions, so there is no “standard dosage” when seen as interventions. Likewise, these technologies provide a degree of distance and anonymity for subjects both inimical to following up outcomes (we do not know who is using it “in the wild”) and difficult to reproduce in controlled environments (normal conditions of use are very unlike controlled conditions).

These issues may help explain the lack of relatively robust evidence for such interventions when compared to text messaging interventions where dosage (ie, the messages sent and their content) is largely under the control of the researchers and can be standardized (with some variation) across hundreds of participants. We would argue that the very nature of interactive media here undermines the scope for large scale intervention studies.

### The Institutional Problem

More speculatively, we suggest this will imply significant *institutional* hurdles that can be expected to work against the successful publication of research in this area, thereby lowering incentives for research investment.

The most obvious impediment is with regard to what is taken to be good evidence in health care research in general, when combined with the measures problem as described above. Because of the measures problem, it is difficult to conduct a randomized controlled trial of a social media or mobile app intervention that simultaneously approaches the gold standard for evidence in the health sciences and also studies real-world engagement with the intervention. Likewise, because real-world dissemination of apps and social media is user-driven, the capacity for robust recruitment and follow-up methodology is greatly impaired.

This is in stark contrast to text messaging interventions where dosage can be firmly under the control of researchers and delivered directly to recruited test subjects who can be directly contacted for follow-up purposes. The same features of social media and mobile software that give them great ease of use and massive disseminability therefore also make them relatively inimitable to traditional academic research. Social media and mobile software use is too slippery a fish for the standard research nets to catch.

So we can expect there to be a systematic barrier against the production of peer-reviewed publications in this area, which may be acting to de-incentivize much-needed research. This is not to say that evidence itself in this field is not possible and program evaluations are generally expected to be built in to publically funded health promotion projects (ie, to gather evidence regardless of foreseen academic output). Measures of reach and usage for apps and social media can be easily obtained from social media analytics and well-designed software to at least gain an understanding of the degree of uptake. However, even if good “reach” data is obtained, there is still a problem of recruitment for the sake of comparative data: recruiting the app/social media users for assessment of health, knowledge, or behavior status. And randomization into intervention and control groups faces further significant technical hurdles. These are fundamental challenges for both research and evaluation in this area, though there are some encouraging signs that innovative researchers can find ways to approach them in a robust manner [32].

One final piece of evidence in this regard concerns timeframe and turnaround of the research itself. Many of the studies we found were published several years after the initial data collection had been carried out and the systematic reviews found were similarly published at a considerable remove from the most recent publications reviewed in each. The optimistic outlook here is that perhaps this slow process is concealing a coming wave of recent mobile software or social media studies. However, it also further diminishes the value of conducting research in such a rapidly changing field; if not carefully designed for general applicability, by the time the research is published there is a non-trivial chance that the studied intervention is obsolete or that the target audience has moved on.

### Future Research

More research on social media and mobile software interventions is required to establish how effective these interventions are for health promotion purposes and under what conditions they can be made more so. However, there is also reason to think that there are several specific impediments to research in this area, which themselves call for further research.

The task for evidence-seeking research and evaluation projects in consumer-focused eHealth intervention is a challenging one, consisting of three distinct requirements: (1) incorporate all useful measures of reach, behavior, and use, (2) make a connection between these measures and (some measure of) health outcomes or health-relevant behavior, and (3) conduct this research in a manner that is comprehensible and rapidly disseminable for peer review.

At the abstract level, we have suggested that there are three areas that will need to be addressed. The process of doing this may require deeper health researcher engagement with the relevant literature in media studies, information technology, and eCommerce, to clarify the concepts and methods involved, converge more upon a common technical language, and develop evidential standards for the use of software analytics and other evidence not commonly found in health and medical research. This might be seen as a need for relevant research in knowledge translation and implementation: to better understand how to translate the principles of commercial success in social media and mobile software into effective health promotion interventions, and how to better integrate these methods into health research.

At a more applied level, there is greater urgency for researchers and health promotion professionals to cooperate in the evaluation of social media and mobile software interventions. As we found, there are numerous projects in Australia alone that could constitute valuable experiments if the right data were also able to be gathered and the results made applicable for peer review and public dissemination.

Concerning the limitations of this scoping study, while we are satisfied that we have sampled a suitable cross-section of research literature and existing projects for our particular purposes, our focus on specific health topics make it likely we have missed some relevant studies and initiatives. Future studies into similar literature (eg, investigating different target conditions) would be advised to carefully consider search strategies as used in the many systematic reviews in eHealth now available. Our survey of Indigenous health programs was also limited to examining publically available information and will not have captured evaluation activity that is underway or planned.

### Indigenous Australia

Finally, we conclude by returning to consider our original framing question: how might social media and mobile technologies best be leveraged for maximum reach and best health outcomes among Indigenous populations? To answer this question would be to consider one further issue inherent to the interactive and collaborative nature of social media and mobile technologies: the unpredictable and culturally-specific nature of their use.

The adoption of an online social media platform is a paradigmatically chaotic social process and appears to occur when one becomes the dominant available space for an already socially self-identifying population to move (at least some of) their social networking activities into. It is well understood that the uptake of social media platforms and mobile software has varied widely according to language, culture, and demographics, and for a variety of sometimes surprising reasons. Usage is driven by the utility of social connections and coherence, but unexpected contingencies often seem to be deciding factors, which explains, for example, why Facebook dominates in English-speaking countries, while it was Orkut (an early attempt at a social network by Google) that rose to early dominance in Brazil and India [37]. Among some Indigenous communities in the Northern Territory, a similar contingency occurred with the rapid uptake of “Divas Chat”, a minimal messaging and social network platform attached to Telstra pre-paid services, but which offers greater reliability in such remote communities [38,39].

Observations such as this (and consideration of the cultural uniqueness of rural and remote Indigenous communities) raise several questions relevant to researchers looking at this area. Of particular interest here is: do culturally-specific modes and organizing principles around “offline” social networking produce specific ways of engaging with online social media and/or absorbing the content therein?

Addressing considerations such as these is certainly not just the domain of health and medical Internet researchers; they touch on how the introduction of modern media influences a community and how technology coexists with culture. Therefore, a full understanding of what might work, and how, may require cross-disciplinary research including the contributions from, for example, cultural researchers and social anthropologists—and, of course, the communities themselves.

### Conclusions

Social media and mobile software interventions are already being used for health promotion and appear to hold great promise, especially for Indigenous or other traditionally underserved populations. However, the current evidence for their effectiveness or health benefit is sparse and mixed. This lack of evidence should not necessarily be seen as an indictment, as it is perhaps to be expected given the mercurial and elusive nature of these interventions and how target populations engage with them. Nevertheless, intervention projects being developed in this area, no matter how well thought out or enthusiastically pursued, cannot be described as entirely evidence-based given the current state of the evidence.

However, for health promotion, there is no real alternative but to engage with social media and mobile software technology, as these forms of online interaction are becoming increasingly ubiquitous at the expense of more traditional media. This means that a more thorough and professional understanding of these technologies will increasingly be called for, at the level of the technologies themselves, but also with regard to how they are in turn engaged with by the specific populations of interest.

## Abbreviations

RCT: randomized controlled trial
SMS: short message service
